# Dietary (Poly)phenol Intake, Inflammatory Biomarkers, and Mild Cognitive Impairment in Italian Adults

**DOI:** 10.3390/antiox15091181

**Published:** 2026-09-17

**Authors:** Margherita Grasso, Francesca L’Episcopo, Giuseppe Toscano, Evelyn Frias-Toral, Stefano Muratore, Maria Angela Tripodi, Sabrina Musso, Veronica Bentivegna, Lucrezia Costanzo, Giusi Fatati, Chiara Mascali, Giorgia Serena Gullotta, Raynier Zambrano-Villacres, Lisandra León Brizuela, Fabio Galvano, Giuseppe Grosso, Justyna Godos, Raffaele Ferri, Giuseppe Lanza, Filippo Caraci

**Affiliations:** 1 Oasi Research Institute-IRCCS, 94018 Troina, Italysmusso@oasi.en.it (S.M.); giorgia.gullotta@unict.it (G.S.G.); rferri@oasi.en.it (R.F.); giuseppe.lanza1@unict.it (G.L.); 2 School of Medicine, Universidad Católica de Santiago de Guayaquil, Av. Pdte. Carlos Julio Arosemena Tola, Guayaquil 090615, Ecuador; 3 Facultad de Ciencias de la Salud y Desarrollo Humano, Universidad Ecotec, Km.13.5 Samborondón, Samborondón EC092302, Ecuador; razambrano@ecotec.edu.ec; 4 Universidad Internacional Iberoamericana, Campeche 24560, Mexico; 5 Universidade Internacional do Cuanza, EN250 Cuito, Bié, Angola; 6 Fundación Universitaria Internacional de Colombia, 111321 Bogotá, Colombia; 7 Department of Biomedical and Biotechnological Sciences, University of Catania, 95123 Catania, Italy; 8 Department of Surgery and Medical-Surgical Specialties, University of Catania, 95123 Catania, Italy; 9 Department of Drug and Health Sciences, University of Catania, 95123 Catania, Italy

**Keywords:** (poly)phenols, anthocyanins, inflammation, cognition, brain, mild cognitive impairment, diet

## Abstract

Background: Dietary (poly)phenols have been proposed to protect against age-related cognitive decline through antioxidant, anti-inflammatory, and neuroprotective mechanisms. However, evidence specifically addressing mild cognitive impairment (MCI) remains limited. This study investigated the association between habitual dietary (poly)phenol intake, cognitive status, and circulating inflammatory biomarkers in older adults. Methods: In this cross-sectional study, Italian adults aged ≥65 years attending a memory clinic underwent standardized neuropsychological assessment for MCI diagnosis. Habitual dietary intake was assessed using a validated food frequency questionnaire, and dietary (poly)phenol intake was estimated using the Phenol-Explorer database. Plasma transforming growth factor-β1 (TGF-β1) and tumor necrosis factor-α (TNF-α) concentrations were measured by ELISA. Logistic regression analyses were performed to evaluate associations between quartiles of (poly)phenol intake and MCI after adjustment for energy intake, age, sex, education, and smoking status. Results: Participants without MCI reported significantly higher intakes of total (poly)phenols and several subclasses, including flavonoids, anthocyanins, flavanones, flavones, hydroxycinnamic acids, and lignans. Higher intakes of flavanones, flavones, lignans, hesperetin, naringenin, lariciresinol, matairesinol, and pinoresinol were positively associated with cognitive performance. Individuals in the highest quartile of total (poly)phenol intake had substantially lower odds of MCI (adjusted OR = 0.08; 95% CI: 0.01–0.62), with similar inverse associations observed for flavonoids (OR = 0.16; 95% CI: 0.02–0.92), lignans (OR = 0.18; 95% CI: 0.04–0.89), flavones (OR = 0.18; 95% CI: 0.04–0.87), naringenin (OR = 0.19; 95% CI: 0.04–0.92), lariciresinol (OR = 0.18; 95% CI: 0.04–0.89), matairesinol (OR = 0.19; 95% CI: 0.04–0.93), and pinoresinol (OR = 0.16; 95% CI: 0.03–0.82), when compared to the lowest quartile. Total (poly)phenol intake was not associated with circulating inflammatory biomarkers, although biochanin A and caffeic acid were inversely associated with TNF-α concentrations. Conclusions: Higher habitual intake of selected dietary (poly)phenols, particularly flavonoids and lignans, was associated with a lower likelihood of MCI and better cognitive performance in older adults. However, the absence of consistent associations with inflammatory biomarkers does not support inference regarding an anti-inflammatory mechanism. Given the observational design, small sample size, and multiple statistical comparisons, these findings should be considered exploratory and hypothesis-generating. Prospective studies and randomized controlled trials are needed to confirm these associations and clarify the underlying biological pathways.

## 1. Introduction

By 2050, the global population aged 60 years and older is projected to exceed 2.1 billion individuals, accounting for more than 21% of the world’s population, and reflecting sustained declines in fertility and continued gains in life expectancy [1]. As a consequence, the absolute burden of cognitive impairment rises rapidly. Currently, dementia has been estimated to affect approximately 55 million individuals worldwide and is projected to increase to 78 million by 2030 and 139 million by 2050 [2]. Importantly, late-life cognitive impairment is etiologically heterogeneous [3]. In addition to Alzheimer’s disease (AD), vascular cognitive impairment and dementia represent a major contributor to the global burden of cognitive decline, either as a primary diagnosis or in mixed forms with Alzheimer’s pathology [4]. Beyond dementia, milder syndromes are highly prevalent; in particular, recent meta-analyses suggest that about 15% of community-dwelling adults aged 50 years and older meet criteria for mild cognitive impairment (MCI), a clinical condition characterized by objective cognitive decline that exceeds what is expected for an individual’s age and educational level, while largely preserving independence in daily functioning, underscoring a substantial at-risk population and continuum from normal aging to dementia [5]. Together, rapid population aging and the increasing prevalence of MCI predict a significant growth in dementia cases and care needs over coming decades, with disproportionate impacts in low- and middle-income countries [6].

Lifestyle habits are considered of paramount importance in shaping the risk of cognitive decline in older adulthood [7]. Among them, diet has been the focus of recent research to assess valid risk or protective factors to support a healthy aging brain [8]. Observational studies comprehensively show that higher intake of plant-based foods, such as fruit and vegetables, has been associated with better cognitive health in all ages and lower risk of cognitive decline [9,10,11]. Consistently, dietary patterns rich in plant-derived foods have been shown to be potentially associated with lower risk of cognitive decline and dementia [12,13,14]. Among the most studied, adherence to the Mediterranean diet, rich in vegetables, fruits, legumes, whole grains, and extra-virgin olive oil, has been linked to reduced incident dementia [15]. The MIND diet (a Mediterranean-DASH hybrid emphasizing leafy greens, berries, nuts, olive oil, and fish while limiting red/processed meats and sweets) has shown robust observational associations with slower cognitive decline and lower Alzheimer’s disease incidence, and newer population-based analyses continue to report reduced dementia risk with higher MIND scores [16]. Evidence for the DASH pattern (designed for blood-pressure control) also points to protection against cognitive decline in several cohorts and quantitative reviews [17]. Although evidence from randomized controlled trials (RCTs) resulted in limited findings [18], multidomain trials suggest that integrated approaches including vascular risk management and cognitive training may lead to significant benefits on global cognition, supporting diet as a key component of effective prevention strategies [19,20].

Plant-based dietary patterns have been hypothesized to potentially exert positive effects on brain health through a variety of mechanisms, including but not limited to the variety of compounds exerting anti-inflammatory actions [21,22]. In fact, plant foods are characterized by high content in phytochemicals and bioactive compounds, such as (poly)phenols [23]. In recent decades, higher dietary intake of (poly)phenols has been associated with lower risk of numerous chronic conditions, such as cardiovascular diseases, hypertension, certain types of cancers, and mortality [24]. Notably, a recent meta-analysis of population studies pointed out that higher dietary intake of flavonoids, a major class of (poly)phenols, is inversely associated with cognitive decline and likelihood of developing dementia [25]. Several clinical intervention trials also provided some evidence that inclusion of (poly)phenol-rich foods may improve cognitive function in older adults [26,27,28,29]. Given the potential role of vascular health in cognitive health [30], evidence from human studies is mechanistically supported by numerous preclinical results demonstrating the direct and indirect neuroprotective effects of (poly)phenols, including modulation of neuro-inflammation, mitochondrial function, regulation of cerebral blood flow, and modulation of neurogenesis and synaptic plasticity [31,32]. However, information on MCI is generally scarcely reported. Hence, further evidence is needed to better understand the role of these bioactive compounds in the prevention and potential mitigation of early-stage cognitive decline. Therefore, the aim of this study was to strengthen the evidence on the association between habitual (poly)phenol intake and MCI in a sample of older Italian adults.

## 2. Materials and Methods

### 2.1. Study Design and Population

This cross-sectional study included consecutive patients evaluated at the Geriatrics Unit of the IRCCS Oasi Research Institute in Troina, Italy. Eligible participants were aged 65 years or older who underwent a visit due to subjective memory impairment and were later diagnosed with subjective cognitive decline, isolated memory impairment, MCI, or mild vascular cognitive impairment. In order to be included, patients had to be autonomous and not report a history of isolation. Individuals were excluded if they had a diagnosis of dementia due to causes other than MCI, dementia associated with other neurological disorders, or major psychiatric illnesses, including major depressive disorder or psychotic disorders. Given the exploratory nature of the study, no a priori sample size calculation was performed.

Clinical diagnoses were established by the Department for Cerebral Involution (Psychology Unit) according to the criteria of the Diagnostic and Statistical Manual of Mental Disorders, Fifth Edition (DSM-5). Among approximately 1000 individuals referred for evaluation of subjective memory complaints, 908 were excluded because they did not satisfy the eligibility criteria, primarily owing to confirmed or suspected alternative neurological or psychiatric conditions. Consequently, 92 participants were considered eligible, and all agreed to participate in the study (Supplementary Figure S1).

The study protocol complied with the principles of the Declaration of Helsinki and Good Clinical Practice guidelines. Ethical approval was granted by the Ethics Committee of the IRCCS Oasi Research Institute, Troina, Italy (CEL-IRCCS OASI/10-04-2025/04; Verbale n. 9 del 19/10/2023, Local Project Code: T5-AN-11). Written informed consent was obtained from all participants before enrollment.

### 2.2. Background Characteristics

Baseline demographic, lifestyle, and clinical information was collected through structured interviews and standardized clinical assessments. Variables considered as potential confounders included age, sex, and smoking status. These covariates were selected based on previous evidence supporting their association with both dietary habits and cognitive outcomes.

### 2.3. Dietary Assessment

Habitual dietary intake was evaluated using a previously validated food frequency questionnaire (FFQ) [33], administered by trained personnel. Participants were asked to report their usual consumption frequency for 110 food and beverage items during the previous year. For each item, intake frequency was recorded using nine predefined response options ranging from “never or almost never” to “four to five times per day,” allowing the assessment of long-term dietary patterns.

### 2.4. Dietary (Poly)phenol Estimation

The methodology used to estimate dietary (poly)phenol intake has been described previously in detail [34]. Foods with no detectable (poly)phenol content were excluded, resulting in 75 food items being retained for the analysis. Standard portion sizes incorporated into the FFQ were combined with reported consumption frequencies to calculate the average daily intake of each food and beverage item, expressed in grams or milliliters per day. The Phenol-Explorer database [35,36] was used to obtain the mean concentrations of total (poly)phenols, their principal subclasses, and selected individual compounds for all relevant FFQ items. For this purpose, (poly)phenol-specific retention factors were obtained from the Phenol-Explorer module, reporting changes in polyphenol concentrations associated with cooking and food-processing procedures. For FFQ items representing composite food categories or potentially matching multiple entries in Phenol-Explorer, the polyphenol composition was weighted according to previously used standardized food composition proportions [34]. (Poly)phenol intake estimates were primarily based on analytical data generated by reverse-phase high-performance liquid chromatography (RP-HPLC) for individual phenolic compounds. For foods such as cereals, beans, and walnuts, in which a proportion of (poly)phenols is not efficiently recovered under conventional extraction conditions, HPLC data obtained following hydrolysis were used instead. Daily intake of major (poly)phenol classes (flavonoids, phenolic acids, lignans, stilbenes) was estimated by multiplying their concentration in each food by the corresponding daily consumption. Total (poly)phenol intake was estimated as the sum of all (poly)phenol classes. Intakes of relevant (poly)phenol subclasses and selected individual compounds were also calculated.

### 2.5. Cognitive Assessment and Diagnosis of MCI

Global cognitive performance was assessed using a standardized neuropsychological battery that included the Mini-Mental State Examination (MMSE) [21,22] and the Montreal Cognitive Assessment (MoCA) [37].

The MMSE is a 30-point clinician-administered screening instrument that evaluates global cognitive status across several domains, including orientation, memory, attention and calculation, language, and visuoconstructive abilities. Participants completed a series of standardized verbal and written tasks, with higher total scores indicating better cognitive functioning. Interpretation of MMSE scores was based on normative values adjusted for age and educational level.

The MoCA, also scored from 0 to 30, was administered to improve the detection of subtle cognitive deficits, particularly in executive and attentional domains. The test evaluates visuospatial and executive functions, naming, attention, language, abstraction, delayed recall, and orientation through a combination of drawing tasks, verbal responses, and other structured exercises. Following standard recommendations, one additional point was assigned to participants with 12 years of education or less. Higher scores indicate better cognitive performance, whereas values below established normative thresholds suggest cognitive impairment.

The diagnosis of MCI was established according to internationally recognized criteria consistent with the DSM-5. Participants were classified as having MCI when they exhibited: (i) subjective cognitive complaints reported by the participant or an informant; (ii) objective impairment in one or more cognitive domains based on age- and education-adjusted normative data; (iii) preservation of functional independence in everyday activities; and (iv) absence of dementia. Based on Petersen’s classification [38], patients with predominant impairment in one or more memory domains were categorized as having amnestic MCI (n = 37), whereas those presenting deficits mainly involving executive function, language, or visuospatial abilities were classified as non-amnestic MCI (n = 55). Diagnostic classification integrated MMSE and MoCA performance with clinical evaluation and functional assessment using the modified Barthel Index of Activities of Daily Living (BADL) and the Instrumental Activities of Daily Living (IADL) scale.

### 2.6. Psychological Covariates

To account for psychological factors that could influence cognitive performance, participants underwent standardized assessments of depressive symptoms and daytime sleepiness.

Depression was evaluated using both clinician-rated and self-reported instruments. The Hamilton Depression Rating Scale (HDRS) [39] is a clinician-administered questionnaire comprising 17 core items assessing depressive symptoms, including mood, guilt, sleep disturbances, anxiety, psychomotor activity, and somatic complaints. Depending on the item, responses are scored using either 3- or 5-point scales, resulting in total scores ranging from 0 to 52, with higher scores indicating greater symptom severity.

Participants also completed the 15-item short version of the Geriatric Depression Scale (GDS-SF) [40,41], a self-administered questionnaire specifically developed for older adults. Each item requires a dichotomous yes/no response, yielding total scores between 0 and 15, where higher values reflect more pronounced depressive symptoms. The combined use of the HDRS and GDS-SF provided complementary clinician-based and self-reported assessments of mood.

Excessive daytime sleepiness was assessed using the Epworth Sleepiness Scale (ESS) [42], an eight-item self-administered questionnaire evaluating the likelihood of falling asleep during common daily situations. Responses are scored from 0 (“would never doze”) to 3 (“high chance of dozing”), producing a total score ranging from 0 to 24, with higher scores indicating greater daytime sleepiness.

### 2.7. Assessment of Inflammatory Biomarkers

Venous blood samples were collected during the baseline neuropsychological evaluation following standardized procedures described previously [43]. Blood was drawn into EDTA-K2 tubes and processed shortly after collection. Plasma was obtained through an initial centrifugation at 1900 rpm for 10 min, followed by a second centrifugation at 3900 rpm for an additional 10 min to eliminate residual cellular material. Aliquoted plasma samples were subsequently stored at −80 °C until analysis.

Plasma concentrations of transforming growth factor-beta 1 (TGF-β1) and tumor necrosis factor-alpha (TNF-α) were measured using commercially available enzyme-linked immunosorbent assay (ELISA) kits. Active TGF-β1 was quantified in plasma diluted 1:10 using ELISA kit (cat. no. DB100C; R&D systems, Bio-Techne, Minneapolis, MN, USA), while TNF-α concentrations were determined using the corresponding human TNF-α ELISA kit (cat. no. DTA00D; R&D systems, Bio-Techne, Minneapolis, MN, USA), following the manufacturers’ protocols. All samples were analyzed in duplicate, and mean values were used for statistical analyses.

According to the manufacturer’s specifications, the average minimum detectable dose (MDD) for TGF-β1 was 2.38 pg/mL (range: 0.889–5.50 pg/mL). All measured concentrations exceeded the assay’s limit of detection. Similarly, the reported MDD for TNF-α ranged from 2.09 to 6.23 pg/mL (mean: 4.00 pg/mL), and all participant samples yielded concentrations above the detection threshold.

Quality-control samples were included on each ELISA plate to monitor assay performance. Plates were accepte111d only when the standard curve and quality-control measurements met the predefined acceptance criteria. Samples were distributed across assay plates independently of study group to minimize potential plate-related systematic bias. All samples were stored at −80 °C until analysis and underwent no more than two freeze–thaw cycle(s) before measurement. Potential batch effects were assessed by including the same pooled QC sample on each plate, and no relevant plate-to-plate variation was observed. Duplicate measurements were accepted when the coefficient of variation between replicates was ≤20%; samples exceeding this threshold were re-assayed. Standard curves were generated separately for each assay plate using a 4-PL model.

Optical density was measured using a Varioskan LUX Multimode Microplate Reader (Agilent BioTek, Santa Clara, CA, USA) at wavelengths of 450, 540, and 570 nm. Final absorbance values were obtained by subtracting the readings at 540 or 570 nm from those measured at 450 nm, in accordance with standard ELISA data processing procedures.

### 2.8. Statistical Analysis

Participant characteristics were described according to total (poly)phenol intake. Continuous variables were expressed as means with standard deviations, whereas categorical variables were expressed as frequencies and percentages. Between-group differences were evaluated using Mann–Whitney U (Wilcoxon rank-sum) or Kruskal–Wallis tests for continuous variables (not normally distributed) and chi-square tests for categorical variables, as appropriate. Spearman’s coefficients were calculated to test correlations between continuous variables. Logistic regression analyses were performed to calculate odds ratios (ORs) and 95% confidence intervals (CIs) adjusted for energy intake (model 1) and for known measured potential confounding factors, such as sex, age, educational level, and smoking status (model 2). Adjustment for multiple comparisons was not performed because of the limited sample size. All statistical analyses were conducted using SPSS 29 (SPSS Inc., Chicago, IL, USA). A two-sided *p*-value < 0.05 was considered statistically significant.

## 3. Results

The study included 92 older adults with memory complaints. Participants were categorized in quartiles of dietary (poly)phenol intake. No significant differences were observed between groups with respect to demographic or lifestyle characteristics, including sex, educational level, and smoking status, while individuals reporting higher total (poly)phenol intake were significantly younger than their counterparts (Table 1).

Likewise, clinical measures, including MMSE, MoCA, GDS, HDRS, and ESS scores, did not differ significantly between low and high (poly)phenol intake groups (Table 2).

**Figure 1:**
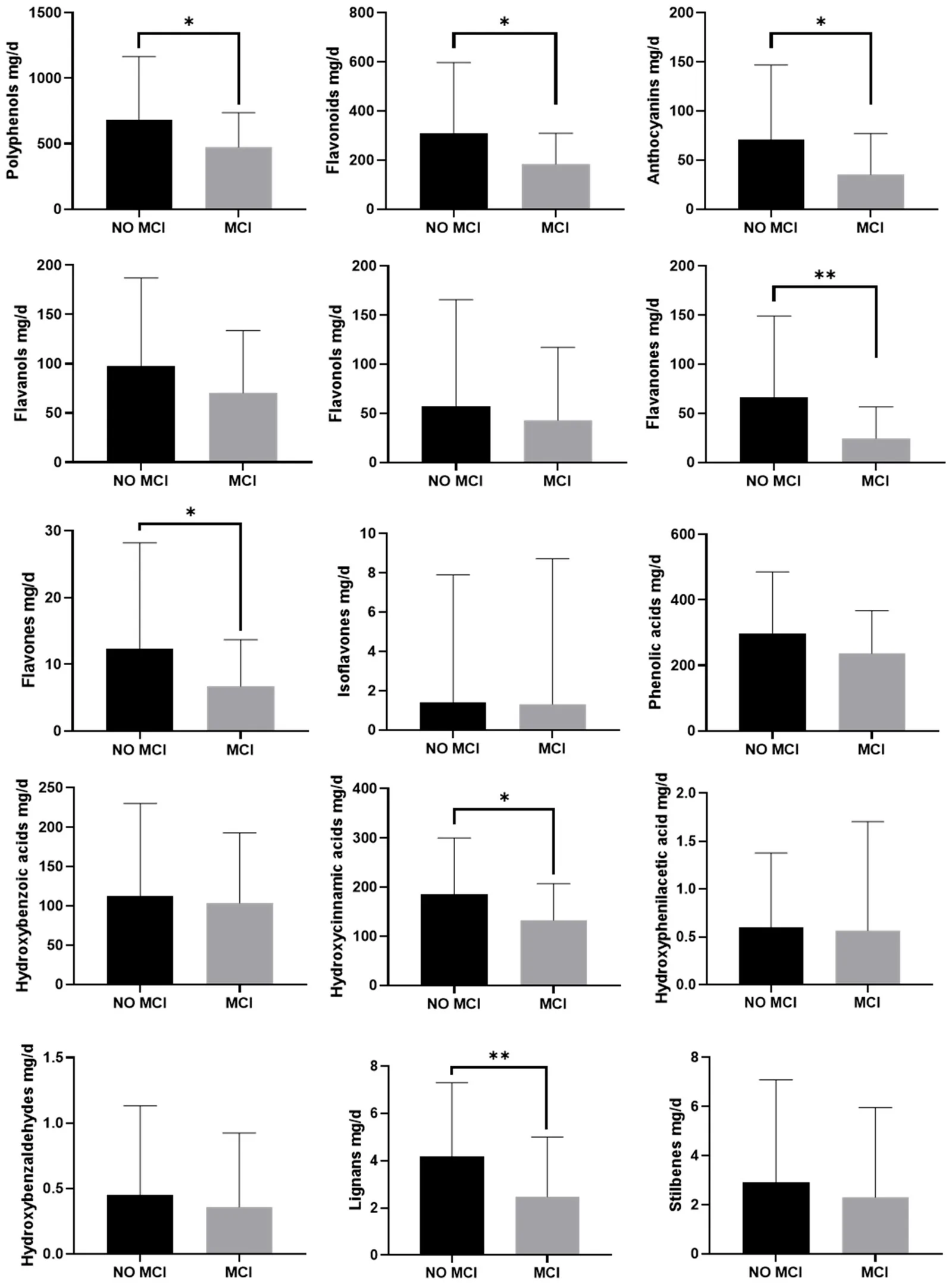
Total and major classes of (poly)phenol intake by clinical and instrumental diagnosis of mild cognitive impairment (MCI). * *p* < 0.05; ** *p* = 0.001.

Comparison according to cognitive status showed that participants without MCI had significantly higher intakes of total dietary (poly)phenols than those with MCI (Figure 1). Among the major (poly)phenol classes, flavonoids, anthocyanins, flavanones, flavones, hydroxycinnamic acids, and lignans were all significantly higher in the non-MCI group, whereas no significant differences were observed for flavanols, flavonols, isoflavones, phenolic acids, hydroxybenzoic acids, hydroxyphenylacetic acids, hydroxybenzaldehydes, or stilbenes (Figure 1).

**Figure 2:**
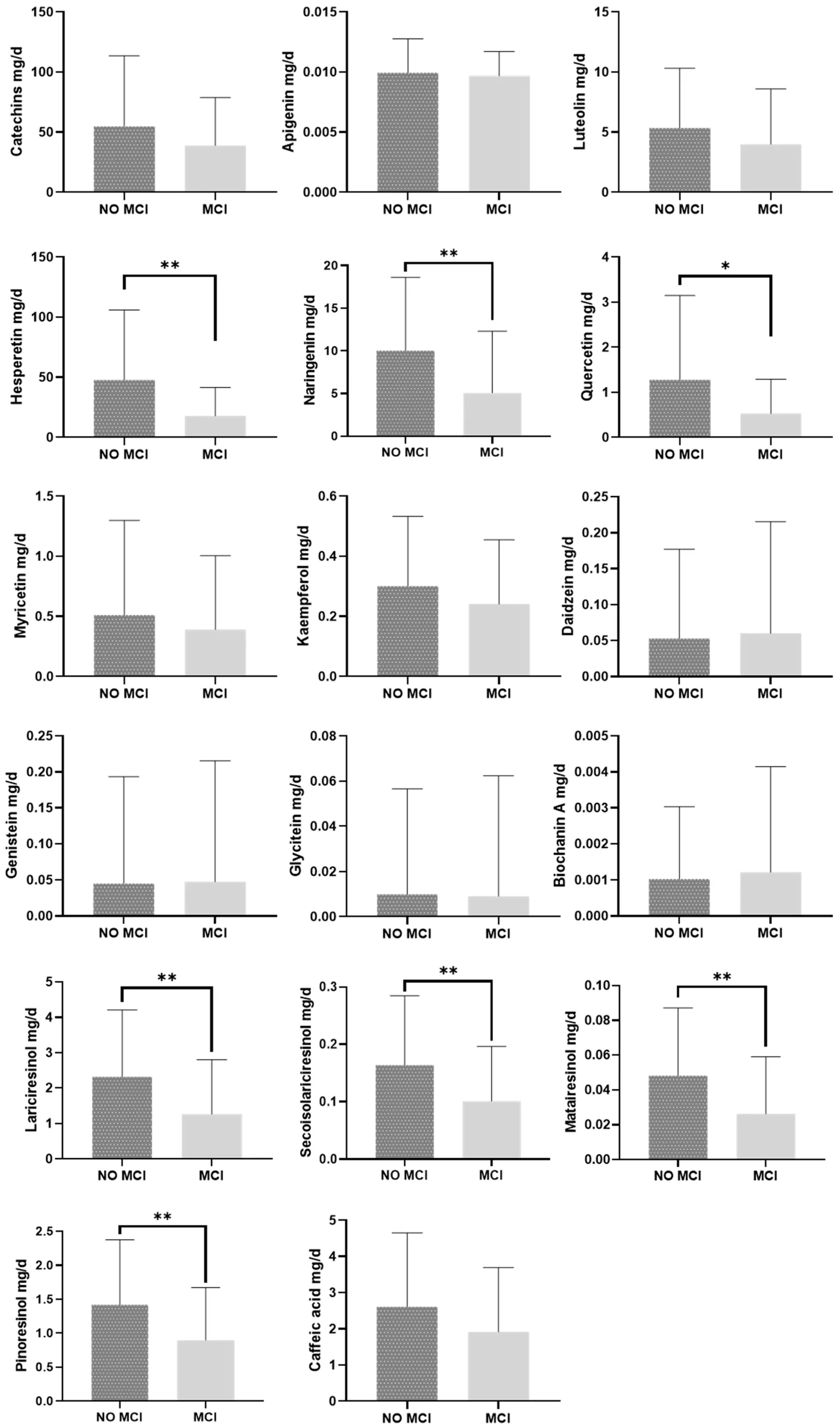
Individual (poly)phenol compound intake by clinical and instrumental diagnosis of mild cognitive impairment (MCI). * *p* < 0.05; ** *p* = 0.001.

Analysis of individual compounds further demonstrated significantly greater intakes of hesperetin, naringenin, quercetin, lariciresinol, secoisolariciresinol, matairesinol, and pinoresinol among participants without MCI (Figure 2). Conversely, intake of catechins, apigenin, luteolin, myricetin, kaempferol, daidzein, genistein, glycitein, biochanin A, and caffeic acid did not differ significantly between MCI groups (Figure 2).

Spearman correlation analysis revealed several significant associations between (poly)phenol intake and cognitive performance (Table 3). MMSE score was significantly correlated with anthocyanin intake (r = 0.214), flavonol intake (r = 0.221), flavanone intake (r = 0.249), hesperetin intake (r = 0.235), and glycitein intake (r = 0.221).

MoCA score showed significant correlations with flavanone intake (r = 0.412), flavone intake (r = 0.370), lignan intake (r = 0.348), luteolin (r = 0.289), hesperetin (r = 0.407), naringenin (r = 0.366), glycitein (r = 0.269), lariciresinol (r = 0.356), secoisolariciresinol (r = 0.331), matairesinol (r = 0.359), and pinoresinol (r = 0.347). Regarding mood and sleep parameters, GDS score was inversely associated with biochanin A intake (r = −0.298), while ESS score was positively associated with daidzein intake (r = 0.233). HDRS score showed inverse correlations with total flavonoid intake (r = −0.222), hydroxybenzaldehydes (r = −0.217), stilbenes (r = −0.222), myricetin (r = −0.268), kaempferol (r = −0.321), and caffeic acid (r = −0.303). Notably, most coefficients are small in magnitude and reflect rather modest correlations. No significant correlations were observed between total dietary (poly)phenol intake and MMSE, MoCA, GDS, ESS, or HDRS scores.

A significant correlation between TGF-β1/TNF-α ratio plasmatic level and MMSE score in MCI patients (r = −0.487) as well as between TGF-β1/TNF-α ratio and MoCa score (r = −0.4614) was found, suggesting that the measurement of this proposed biomarker could reflect in the periphery an inflammatory response related to the worsening cognitive functioning.

Correlation analyses with inflammatory biomarkers demonstrated no significant associations between total (poly)phenol intake and circulating TGF-β, TNF-α, or the TGF-β:TNF-α ratio (Table 4). Among the major (poly)phenol classes, only flavanol intake was inversely correlated with TGF-β concentrations (r = −0.211). At the individual compound level, biochanin A intake was inversely associated with TNF-α (r = −0.306) and positively associated with the TGF-β:TNF-α ratio (r = 0.245). Similarly, caffeic acid intake was inversely correlated with TNF-α concentrations (r = −0.298). Notably, in this analysis, the magnitude of the correlation coefficients was confirmed to be rather weak. No other significant associations between (poly)phenol subclasses or individual compounds and inflammatory biomarkers were identified.

The association between total and major classes of (poly)phenols and MCI status is reported in Table 5. Individuals in the highest quartile of total (poly)phenols (OR = 0.08, 95% CI: 0.01, 0.62), flavonoids (OR = 0.16, 95% CI: 0.02, 0.92), and lignans (OR = 0.18, 95% CI: 0.04, 0.89) were less likely to have MCI compared to those in the lowest quartile of intake, even after adjustment for potential confounding factors. Among individual flavonoid classes, flavones showed the most promising associations with MCI status (for the highest vs. the lowest quartile of intake, OR = 0.18, 95% CI: 0.04, 0.87; Table 5).

Table 6 shows the association between individual (poly)phenols and MCI status. Among the main flavonoid compounds, individuals in the highest quartile of flavanone naringenin (OR = 0.19, 95% CI: 0.04, 0.92) and individual lignans lariciresinol (OR = 0.18, 95% CI: 0.04, 0.89), matairesinol (OR = 0.19, 95% CI: 0.04, 0.93), and pinoresinol (OR = 0.16, 95% CI: 0.03, 0.82) intake had lower odds of having MCI, when compared to the lowest quartile of intake.

## 4. Discussion

Overall, the findings presented in this study indicate that participants without MCI reported greater consumption of several flavonoid subclasses and lignans. Although total dietary (poly)phenol intake was not correlated with cognitive scores or inflammatory biomarkers, specific (poly)phenol classes and compounds (particularly flavanones, lignans, hesperetin, naringenin, biochanin A, and caffeic acid) showed significant correlation with cognitive performance, depressive symptoms, and markers of systemic inflammation. Finally, participants consuming more total (poly)phenols and, in particular, flavonoids and lignans, were less likely to have MCI. Notably, most groups of compounds (with the exception of isoflavones) showed an inverse association with MCI status, yet did not reach statistical significance.

The present findings are broadly consistent with some previous epidemiological evidence reporting associations between dietary flavonoid intake and cognitive health [25], although the null and inconsistent associations observed in the present study warrant cautious interpretation. In the present study, we found that participants without MCI reported significantly higher intakes of total (poly)phenols, flavonoids, anthocyanins, flavanones, flavones, hydroxycinnamic acids, and lignans, while flavanones and flavones exhibited the strongest positive correlations with global cognitive performance, particularly MoCA scores. Significant associations with MCI status were found for flavonoids (among which, flavones exhibited the strongest association) and lignans. Previous epidemiological studies conducted on large cohorts showed an inverse relation between flavones and flavone-rich foods and dementia risk [44,45] and subjective cognitive decline [46,47], although not unequivocally demonstrated [48]. Similar findings from cohort studies showed that lignan intake was associated with favourable cognitive functions [49,50,51,52]. The correlation between anthocyanin intake and cognitive measurements is in line with recent comprehensive evidence in the literature showing a potential effect of anthocyanin supplementation (i.e., through berry fruit derivatives) on cognitive function [26]. Similarly, other comprehensive overviews of the scientific literature reported effects for dietary intervention studies with sources of flavanones (i.e., citrus fruit) [53,54], as well as observational studies on individual compounds, such as hesperetin and naringenin, reporting associations between citrus flavanones and cognitive outcomes, with endothelial function, cerebral perfusion, and neuroinflammatory pathways proposed as possible mechanisms [55,56]. Likewise, similar effects toward better cognitive brain functions have been observed for coffee [57], as well as for main sources of coffee-derived chlorogenic acids, such as hydroxycinnamic acids [58], as well as previous reports suggesting beneficial associations between dietary lignans and risk of dementia [59]. However, unlike most previous investigations, our study specifically examined individuals with clinically and instrumentally diagnosed MCI rather than cognitive performance in community-dwelling populations. While most available cohort studies have focused on incident dementia or age-related cognitive decline [44,46], only a limited number of investigations have been specifically conducted on MCI populations. A recent case–control study reported that higher dietary flavonoid intake was associated with lower odds of MCI in older adults, with flavonols and isoflavones showing the strongest associations, providing further support for the relationship observed in the present study [60]. Among other observational studies, a retrospective report showed that MCI patients who consumed more cocoa (poly)phenols (flavan-3-ols) exhibited a lower rate of worsening in cognition over time [61].

Although biological mechanisms linking dietary (poly)phenols with inflammatory pathways have been proposed, the present study provides limited support for such a relationship, as total (poly)phenol intake was not associated with the measured inflammatory biomarkers. (Poly)phenol supplementation has been shown to decrease blood IL-6 as well as enhance cognitive function, while evidence on TNF-α remains contrasting [62]. Preclinical studies extensively describe an attenuation of neuroinflammation as a major mechanism for several (poly)phenol compounds [63,64]. Preclinical studies show anti-inflammatory action for individual (poly)phenol classes, including flavones [65,66] and lignans [67,68], which ameliorate age-related conditions in animal models. Laboratory research shows that flavones may inhibit astrocyte overactivation and neuroinflammation (TNF-α, IL-1β, IL-6, NO, COX-2, and iNOS protein), and decrease the expression of endoplasmic reticulum stress markers in brain tissues [69]. Similarly, lignans mitigated cognitive dysfunction and reduced p-Tau as well as neuronal loss in Alzheimer’s disease model mice [70], repressing the mitogen-activated protein kinase (MAPK) and nuclear factor-κB (NF-κB) pathways through upregulating AMPK [71], upregulating the ratio of Bcl-2/Bax, downregulating cytochrome c and cleaved caspase-3 expressions, and reducing the expression of Toll-like receptor 4 (TLR4) [72]. Among other compounds that showed correlation with cognitive functioning, experimental studies have demonstrated anti-inflammatory and neuroprotective properties of biochanin A, mainly through inhibition of NF-κB signaling, attenuation of microglial activation, and reduction in pro-inflammatory cytokine production [73]. Therefore, our finding that higher dietary biochanin A intake was associated with lower circulating TNF-α concentrations and a higher TGF-β/TNF-α ratio should be considered novel and hypothesis-generating. Animal studies also support the mechanistic action of naringin against oxidative stress-induced neurobehavioral disorders and cognitive dysfunction [74]. Similarly, the inverse association between caffeic acid intake and TNF-α is supported by experimental evidence demonstrating antioxidant and anti-inflammatory actions of hydroxycinnamic acids [75]. Taken together, these observations identify selected compound-specific associations that warrant further investigation; however, given the absence of an association between total (poly)phenol intake and inflammatory biomarkers, the present findings do not establish an anti-inflammatory or neuroprotective mechanism. Although the cross-sectional design of our study does not allow causal conclusions, and total (poly)phenol intake was not significantly associated with circulating inflammatory biomarkers, several (poly)phenol subclasses and individual compounds associated with circulating pro- and anti-inflammatory cytokines may be interconnected with specific bioactive compounds consumed rather than total (poly)phenol intake alone. Nonetheless, the isolated associations observed for selected subclasses and individual compounds should be interpreted cautiously given the number of comparisons performed and require replication in independent studies.

Interconnections between dietary (poly)phenols, the gut microbiota, and brain function have been proposed as potential pathways linking diet with cognitive health across the lifespan [76]. Recent research suggests that (poly)phenols can modulate the composition and activity of the gut microbial ecosystem through a bidirectional communication network and prebiotic effects [77], leading to the generation of bioactive metabolites that may promote brain health [78,79], and potentially mitigating age-related changes in the gut microbiome [80]. (Poly)phenol-derived microbial metabolites can cross the blood–brain barrier or influence peripheral immune and endocrine pathways that impact brain function [76,81,82]. Among the metabolites produced by microbial degradation of (poly)phenols, anthocyanin derivatives have attracted attention for their neuroprotective properties and associations with improved cognitive outcomes in older adults [83]. These compounds may exert antioxidant and anti-inflammatory effects directly in the brain or indirectly via modulation of systemic inflammation, which is increasingly recognized in the pathophysiology of neurodegenerative diseases [84,85]. The composition and functional activity of the gut microbiota play a pivotal role in their metabolism, as many (poly)phenols require microbial conversion to generate bioactive metabolites, resulting in distinct metabotypes among individuals [86]. Additional factors such as age, sex, body composition, genetics, diet, and lifestyle further modulate (poly)phenol metabolism, contributing to the observed variability in circulating metabolites and physiological responses [86,87]. Given that gut microbiota composition was not assessed in the present study, the potential contribution of these pathways remains speculative and should be evaluated directly in future studies.

The present study has several strengths that are worth addressing. First, the simultaneous inclusion of (poly)phenol intake, a comprehensive neuropsychological assessment, and circulating inflammatory biomarkers allowed exploratory examination of relationships across these domains. Another important strength is the clinical characterization of cognitive status. The diagnosis of MCI was established through standardized neuropsychological evaluation using validated instruments, rather than relying solely on self-reported cognitive complaints or screening questionnaires. This reduces outcome misclassification and strengthens the validity of the observed associations. Similarly, the FFQ was not self-administered, but trained personnel collected the information and supported the patients at any moment during data collection. Moreover, limited evidence exists in the scientific literature regarding well-characterized samples including patients with MCI and measurements of circulating inflammatory biomarkers.

However, the findings of this study should be interpreted in light of several limitations. First, the cross-sectional design does not allow the establishment of temporal or causal relationships. Reverse causation also cannot be excluded. Participants with better cognitive status may have maintained healthier dietary habits, including greater consumption of (poly)phenol-rich foods, whereas individuals with cognitive impairment may have experienced changes in food choice, dietary variety, or dietary reporting. Second, although the analyses accounted for a range of potential covariates known to be biologically related to the patient-disease risk pair, residual confounding cannot be excluded. In particular, unmeasured factors potentially associated with dietary patterns may have influenced the observed associations. Moreover, the potential contribution of other dietary components correlated with (poly)phenol-rich foods cannot be fully disentangled, raising the possibility that some associations attributed to (poly)phenols may partly reflect the broader nutritional composition and overall quality of the diet. Also, diet may affect weight status and metabolic disturbances, which could be other residual confounders unmeasured. Another limitation concerns the instruments used to collect dietary information, as they may suffer from recall bias, especially in light of the observed memory problems. Finally, diet may be related to socioeconomic status, which in turn may affect access to care and ultimately health status. In addition, inflammatory biomarkers were measured at a single time point and may therefore not adequately reflect long-term inflammatory status or intra-individual biological variability. Moreover, the wide confidence intervals suggest limited precision and findings should be interpreted cautiously considering the relatively small sample size, as it may have reduced statistical power, increased the uncertainty of effect estimates, and limited the ability to detect modest associations. Finally, given the exploratory nature of the study and the number of polyphenol-related exposures investigated, the possibility of chance findings due to multiple comparisons cannot be excluded. Although the examined polyphenol classes, subclasses, and individual compounds are biologically correlated, this dependence does not eliminate the risk of false-positive associations. Considering the number of dietary exposures, biomarkers, and statistical associations examined, the risk of Type I error and false-positive findings due to multiple testing cannot be excluded. Therefore, the observed associations should be interpreted cautiously and require confirmation in larger prospective studies. Notably, inter-individual differences in the absorption, metabolism, and excretion of dietary (poly)phenols, which arise from a complex interplay of genetic, microbial, and physiological factors cannot be accounted for, consequently, underscoring the need for personalized approaches that consider genetic, microbial, and environmental determinants. Accordingly, the observed associations should be regarded as exploratory and interpreted in the context of these methodological and statistical limitations. Among other limitations, the measurement of inflammatory biomarkers at a single time point may not fully capture chronic inflammatory status. Ultimately, the relatively small sample size may have limited statistical power.

## 5. Conclusions

This study provides evidence that higher habitual intake of specific dietary (poly)phenols is associated with a lower likelihood of MCI among older Italian adults. Participants without MCI reported significantly greater consumption of total (poly)phenols, particularly flavonoids, anthocyanins, flavanones, flavones, hydroxycinnamic acids, and lignans. While total (poly)phenol intake was not associated with cognitive performance or circulating inflammatory biomarkers, several individual (poly)phenol subclasses and compounds, including flavanones, lignans, hesperetin, naringenin, biochanin A, and caffeic acid, were positively associated with cognitive function and, in some cases, with lower systemic inflammation. Although the overall magnitude of correlations and associations was relatively weak, these findings suggest that the association between dietary (poly)phenol intake and cognitive status may depend more on the specific bioactive compounds consumed rather than on total (poly)phenol intake alone. However, albeit some correlations with inflammatory biomarkers have been reported, the overall mechanistic process through anti-inflammatory pathways is not conclusive, while this study rather provides exploratory associations requiring confirmation. Moreover, the cross-sectional design, relatively small sample size, and reliance on self-reported dietary data preclude causal inference. Further prospective cohort studies and randomized controlled trials incorporating comprehensive biomarker assessment and gut microbiota characterization are warranted to confirm these findings and clarify the mechanisms underlying the relationship between dietary (poly)phenols and cognitive health. If confirmed, increasing the consumption of (poly)phenol-rich foods could represent a practical dietary strategy for promoting healthy brain aging and reducing the risk of cognitive decline in older adults.

## Figures and Tables

**Table 1:** Background characteristics of the study sample by intake of total (poly)phenols.

	(Poly)phenol Intake				
	Q1	Q2	Q3	Q4	*p*-Value
Sex, n (%)					0.588
Male	8 (34.8)	10 (43.5)	12 (52.2)	12 (52.2)	
Female	15 (65.2)	13 (56.5)	11 (47.8)	11 (47.8)	
Age, mean (SD)	73.5 (6.8)	73.8 (6.7)	76.2 (6.6)	69.4 (5.7)	0.006
Educational level, n (%)					0.881
Low	11 (47.8)	11 (47.8)	8 (34.8)	8 (34.8)	
Medium	10 (43.5)	11 (47.8)	13 (56.5)	12 (52.2)	
High	2 (8.7)	1 (4.3)	2 (8.7)	3 (13.0)	
Smoking status, n (%)					0.572
Smoker	6 (27.3)	9 (39.1)	8 (34.8)	10 (47.6)	
Non-Smoker	16 (72.7)	14 (60.9)	15 (65.2)	11 (52.4)	

**Table 2:** Clinical characteristics of the study sample by intake of total (poly)phenols.

	(Poly)phenol Intake				
	Q1	Q2	Q3	Q4	*p*-Value
MMSE, mean (SD)	26.2 (2.9)	27.3 (2.0)	26.0 (3.5)	27.2 (2.7)	0.294
MoCa, mean (SD)	22.8 (5.3)	24.0 (3.2)	21.8 (4.4)	24.0 (4.1)	0.291
GDS, mean (SD)	3.6 (3.4)	2.4 (2.1)	3.2 (3.0)	3.2 (3.4)	0.610
HDRS, mean (SD)	6.2 (3.0)	5.0 (3.9)	5.3 (4.2)	4.7 (3.5)	0.599
ESS, mean (SD)	2.9 (3.0)	3.2 (2.7)	3.0 (2.6)	3.3 (2.7)	0.979

MMSE, Mini-Mental State Examination; MoCa, Montreal Cognitive Assessment; GDS, Geriatric Depression Scale; HDRS, Hamilton Depression Rating Scale; ESS, Epworth Sleepiness Scale.

**Table 3:** Spearman correlation coefficients for total, major classes, and individual (poly)phenols and clinical measures of cognitive status, depressive symptoms, and sleep quality.

	MMSE	Moca	GDS	ESS	HDRS
Total (poly)phenols	0.184	0.119	−0.010	0.091	−0.171
Flavonoids	0.187	0.205	0.055	0.071	−0.222 *
Anthocyanins	0.214 *	0.021	0.109	−0.005	−0.191
Flavanols	0.132	0.029	0.164	0.081	−0.065
Flavonols	0.221 *	0.163	−0.118	0.053	−0.165
Flavanones	0.249 *	0.412 *	−0.101	−0.125	−0.159
Flavones	0.168	0.370 *	0.079	−0.094	−0.182
Isoflavones	0.068	0.038	−0.067	0.213	−0.118
Phenolic acids	0.132	−0.036	−0.044	0.047	−0.079
Hydroxybenzoic acids	0.110	−0.058	−0.034	0.005	−0.072
Hydroxycinnamic acids	0.139	0.015	0.023	0.041	−0.071
Hydroxyphenylacetic acid	0.123	−0.069	−0.019	0.059	−0.212
Hydroxybenzaldehydes	0.030	−0.102	−0.020	0.048	−0.217 *
Lignans	0.143	0.348 *	−0.059	−0.098	−0.134
Stilbenes	0.072	−0.018	0.038	0.044	−0.222 *
Catechins	0.165	0.080	0.152	0.054	−0.036
Apigenin	0.013	0.022	−0.029	−0.202	−0.083
Luteolin	0.120	0.289 **	0.127	−0.105	−0.199
Hesperetin	0.235 *	0.407 **	−0.089	−0.130	−0.148
Naringenin	0.160	0.366 *	−0.080	−0.124	−0.174
Quercetin	0.153	0.118	0.051	0.001	−0.198
Myricetin	0.008	−0.070	−0.086	−0.045	−0.268 *
Kaempferol	0.031	0.052	−0.121	0.033	−0.321 **
Daidzein	0.080	0.046	−0.088	0.233 *	−0.098
Genistein	0.058	0.019	−0.029	0.198	−0.127
Glycitein	0.221 *	0.269 *	0.081	0.215	0.037
Biochanin A	0.019	0.103	−0.298 **	0.042	−0.097
Lariciresinol	0.150	0.356 **	−0.060	−0.089	−0.142
Secoisolariciresinol	0.125	0.331 **	−0.047	−0.075	−0.126
Matairesinol	0.158	0.359 **	−0.077	−0.123	−0.150
Pinoresinol	0.140	0.347 **	−0.059	−0.083	−0.125
Caffeic acid	0.018	0.015	−0.061	0.011	−0.303 **

* denotes *p* < 0.05. ** denotes *p* < 0.001.

**Table 4:** Spearman correlation coefficients for total, major classes, and individual (poly)phenols and biomarkers of inflammation.

	TGF-β	TNF-α	TGF-β:TNF-α Ratio
Total (poly)phenols	0.013	−0.085	−0.038
Flavonoids	−0.090	−0.073	−0.080
Anthocyanins	−0.114	−0.078	−0.127
Flavanols	−0.211 *	−0.008	−0.176
Flavonols	−0.092	−0.011	−0.138
Flavanones	0.113	−0.042	0.042
Flavones	0.168	0.031	0.011
Isoflavones	−0.009	−0.037	−0.017
Phenolic acids	0.010	0.035	−0.139
Hydroxybenzoic acids	0.033	0.166	−0.193
Hydroxycinnamic acids	−0.048	−0.120	−0.074
Hydroxyphenylacetic acid	−0.154	−0.105	−0.096
Hydroxybenzaldehydes	−0.118	−0.160	−0.018
Lignans	0.015	0.012	−0.074
Stilbenes	−0.124	−0.175	−0.024
Catechins	−0.181	0.040	−0.180
Apigenin	0.128	0.095	−0.009
Luteolin	0.118	0.063	−0.054
Hesperetin	0.113	−0.035	0.042
Naringenin	0.009	−0.044	−0.047
Quercetin	0.011	−0.120	0.066
Myricetin	−0.057	−0.217	0.073
Kaempferol	−0.123	−0.127	−0.055
Daidzein	−0.006	−0.060	0.003
Genistein	0.008	−0.011	−0.007
Glycitein	−0.120	−0.102	0.044
Biochanin A	0.113	−0.306 **	0.245 *
Lariciresinol	0.018	−0.013	−0.059
Secoisolariciresinol	−0.019	0.013	−0.099
Matairesinol	−0.010	−0.003	−0.088
Pinoresinol	0.026	0.004	−0.059
Caffeic acid	−0.053	−0.298 **	0.132

* denotes *p* < 0.05. ** denotes *p* < 0.001.

**Table 5:** Association between quartiles of total and major classes of (poly)phenol intake and MCI status.

	(Poly)phenol Intake, OR (95% CI); n (MCI/Non-MCI)			
	Q1	Q2	Q3	Q4
Polyphenols	14/9	8/15	12/11	3/20
Model 1	1	0.34 (0.10, 1.14)	0.70 (0.21, 2.34)	0.10 (0.02, 0.49)
Model 2	1	0.26 (0.06, 1.06)	0.57 (0.17, 2.41)	0.08 (0.01, 0.62)
Flavonoids	14/9	10/13	10/13	3/20
Model 1	1	0.49 (0.15, 1.63)	0.49 (0.15, 1.61)	0.09 (0.02, 0.46)
Model 2	1	0.43 (0.11, 1.67)	0.49 (0.12, 2.05)	0.16 (0.02, 0.92)
Anthocyanins	12/11	10/13	12/11	3/20
Model 1	1	0.72 (0.22, 2.33)	1.03 (0.32, 3.31)	0.15 (0.03, 0.65)
Model 2	1	1.00 (0.25, 4.07)	1.01 (0.26, 3.99)	0.25 (0.05, 1.34)
Flavanols	12/11	9/14	9/14	7/16
Model 1	1	0.69 (0.20, 2.34)	0.66 (0.20, 2.19)	0.45 (0.13, 1.53)
Model 2	1	0.67 (0.17, 2.71)	0.84 (0.21, 3.39)	0.35 (0.07, 1.58)
Flavonols	13/10	10/13	8/15	6/17
Model 1	1	0.61 (0.19, 1.97)	0.42 (0.13, 1.38)	0.31 (0.08, 1.17)
Model 2	1	1.35 (0.34, 5.38)	0.51 (0.13, 2.05)	0.35 (0.07, 1.74)
Flavanones	15/8	11/12	6/17	5/18
Model 1	1	0.51 (0.15, 1.70)	0.20 (0.05, 0.72)	0.16 (0.04, 0.61)
Model 2	1	0.24 (0.05, 1.07)	0.16 (0.04, 0.72)	0.23 (0.05, 1.08)
Flavones	16/7	7/16	8/15	6/17
Model 1	1	0.19 (0.05, 0.70)	0.24 (0.07, 0.85)	0.16 (0.04, 0.63)
Model 2	1	0.13 (0.02, 0.61)	0.19 (0.04, 0.84)	0.18 (0.04, 0.87)
Isoflavones	5/14	12/15	9/12	11/14
Model 1	1	2.33 (0.65, 8.37)	2.54 (0.64, 10.02)	2.68 (0.71, 10.09)
Model 2	1	6.33 (1.22, 32.92)	2.18 (0.40, 11.85)	2.35 (0.47, 11.88)
Phenolic acids	13/10	7/16	9/14	8/15
Model 1	1	0.35 (0.10, 1.19)	0.59 (0.17, 2.03)	0.49 (0.14, 1.72)
Model 2	1	0.59 (0.14, 2.45)	1.05 (0.25, 4.44)	0.67 (0.15, 3.01)
Hydroxybenzoic acids	11/12	8/15	9/14	9/14
Model 1	1	0.55 (0.17, 1.83)	0.75 (0.23, 2.46)	0.79 (0.24, 2.61)
Model 2	1	0.54 (0.13, 2.23)	0.82 (0.21, 3.18)	0.64 (0.15, 2.76)
Hydroxycinnamic acids	13/10	11/12	9/14	4/19
Model 1	1	0.70 (0.22, 2.27)	0.49 (0.15, 1.64)	0.16 (0.04, 0.72)
Model 2	1	1.09 (0.28, 4.32)	0.41 (0.10, 1.67)	0.35 (0.06, 1.94)
Hydroxyphenilacetic acids	11/12	11/12	5/18	10/13
Model 1	1	1.11 (0.34, 3.59)	0.33 (0.09, 1.19)	1.15 (0.33, 4.00)
Model 2	1	0.82 (0.19, 3.44)	0.65 (0.14, 2.91)	2.52 (0.51, 12.39)
Hydroxybenzaldehydes acids	8/10	12/16	9/14	8/15
Model 1	1	0.82 (0.24, 2.79)	0.92 (0.25, 3.32)	0.71 (0.20, 2.54)
Model 2	1	1.39 (0.32, 6.01)	1.05 (0.21, 5.13)	1.24 (0.22, 6.87)
Lignans	15/8	11/12	7/16	4/19
Model 1	1	0.50 (0.15, 1.67)	0.24 (0.07, 0.84)	0.12 (0.03, 0.49)
Model 2	1	0.38 (0.08, 1.69)	0.23 (0.05, 0.99)	0.18 (0.04, 0.89)
Stilbenes	10/13	11/12	9/14	7/16
Model 1	1	1.39 (0.42, 4.60)	1.10 (0.31, 3.91)	0.68 (0.19, 2.39)
Model 2	1	2.61 (0.61, 11.17)	1.25 (0.27, 5.75)	1.16 (0.23, 5.77)

Model 1 was adjusted for energy intake. Model 2 was further adjusted for age (continuous), sex, educational and smoking status. Class/compound rows: n (MCI/non-MCI); - denotes an empty quartile.

**Table 6:** Association between quartiles of individual (poly)phenol compound intake and MCI status.

	(Poly)phenol Intake, OR (95% CI); n (MCI/Non-MCI)			
	Q1	Q2	Q3	Q4
Catechins	12/11	9/14	9/14	7/16
Model 1	1	0.65 (0.20, 2.14)	0.67 (0.20, 2.24)	0.45 (0.13, 1.54)
Model 2	1	0.61 (0.16, 2.37)	0.78 (0.18, 3.33)	0.42 (0.10, 1.84)
Apigenin	4/6	-	23/27	10/22
Model 1	1	-	1.19 (0.29, 4.85)	0.68 (0.15, 3.00)
Model 2	1	-	0.87 (0.17, 4.48)	0.54 (0.10, 3.08)
Luteolin	14/9	9/14	7/16	7/16
Model 1	1	0.42 (0.13, 1.38)	0.29 (0.08, 0.99)	0.33 (0.09, 1.19)
Model 2	1	0.37 (0.09, 1.54)	0.22 (0.05, 0.99)	0.34 (0.07, 1.51)
Hesperetin	15/8	11/12	6/17	5/18
Model 1	1	0.51 (0.15, 1.70)	0.20 (0.05, 0.72)	0.16 (0.04, 0.61)
Model 2	1	0.24 (0.05, 1.07)	0.16 (0.04, 0.72)	0.23 (0.05, 1.08)
Naringenin	14/9	13/10	6/17	4/19
Model 1	1	0.88 (0.26, 2.95)	0.24 (0.06, 0.85)	0.15 (0.03, 0.61)
Model 2	1	0.47 (0.11, 2.03)	0.20 (0.04, 0.86)	0.19 (0.04, 0.92)
Quercetin	11/12	12/11	10/13	4/19
Model 1	1	1.26 (0.39, 4.06)	1.04 (0.30, 3.57)	0.25 (0.06, 0.96)
Model 2	1	1.23 (0.33, 4.58)	1.09 (0.26, 4.50)	0.27 (0.05, 1.40)
Myricetin	-	19/27	6/12	12/16
Model 1	-	1	0.85 (0.25, 2.82)	1.16 (0.44, 3.08)
Model 2	-	1	0.99 (0.24, 4.18)	1.61 (0.41, 6.27)
Kaempferol	12/11	8/15	11/12	6/17
Model 1	1	0.51 (0.15, 1.68)	0.97 (0.29, 3.24)	0.39 (0.10, 1.43)
Model 2	1	0.72 (0.18, 2.86)	1.21 (0.29, 4.97)	0.79 (0.15, 4.17)
Daidzein	5/14	6/10	16/16	10/15
Model 1	1	1.68 (0.40, 7.12)	3.33 (0.94, 11.81)	2.30 (0.61, 8.71)
Model 2	1	3.73 (0.65, 21.42)	4.11 (0.90, 18.72)	2.14 (0.44, 10.46)
Genistein	6/15	5/11	16/15	10/14
Model 1	1	1.12 (0.27, 4.68)	3.21 (0.95, 10.80)	2.22 (0.62, 7.99)
Model 2	1	1.72 (0.33, 9.10)	3.04 (0.72, 12.86)	1.60 (0.35, 7.24)
Biochanin A	-	21/25	6/15	10/15
Model 1	-	1	0.48 (0.16, 1.46)	0.84 (0.31, 2.27)
Model 2	-	1	0.43 (0.12, 1.50)	0.78 (0.23, 2.63)
Lariciresinol	15/8	11/12	7/16	4/19
Model 1	1	0.50 (0.15, 1.67)	0.24 (0.07, 0.84)	0.12 (0.03, 0.49)
Model 2	1	0.38 (0.08, 1.69)	0.23 (0.05, 1.00)	0.18 (0.04, 0.89)
Secoisolariciresinol	14/9	10/13	9/14	4/19
Model 1	1	0.50 (0.15, 1.61)	0.43 (0.13, 1.44)	0.14 (0.03, 0.59)
Model 2	1	0.42 (0.10, 1.76)	0.40 (0.10, 1.61)	0.24 (0.05, 1.18)
Matairesinol	14/9	12/11	7/16	4/19
Model 1	1	0.70 (0.21, 2.29)	0.28 (0.08, 0.99)	0.14 (0.03, 0.58)
Model 2	1	0.34 (0.08, 1.49)	0.26 (0.06, 1.10)	0.19 (0.04, 0.93)
Pinoresinol	16/7	9/14	8/15	4/19
Model 1	1	0.28 (0.08, 0.98)	0.24 (0.07, 0.83)	0.09 (0.02, 0.40)
Model 2	1	0.25 (0.05, 1.11)	0.23 (0.05, 0.97)	0.16 (0.03, 0.82)
Caffeic acid	13/10	8/15	10/13	6/17
Model 1	1	0.42 (0.13, 1.41)	0.64 (0.19, 2.14)	0.31 (0.08, 1.25)
Model 2	1	0.38 (0.09, 1.57)	0.77 (0.18, 3.21)	0.51 (0.08, 3.09)

Model 1 was adjusted for energy intake. Model 2 was further adjusted for age (continuous), sex, educational and smoking status. - denotes an empty quartile.

## Data Availability

The data presented in this study are available upon reasonable request from the corresponding author due to confidentiality regulation.

## Supplementary Materials

The following supporting information can be downloaded at: https://www.mdpi.com/article/10.3390/antiox15091181/s1, Supplementary Figure S1. Study selection process.

## Abbreviations

The following abbreviations are used in this manuscript:

AD	Alzheimer’s disease
AMPK	AMP-activated protein kinase
BADL	Barthel Index of Activities of Daily Living
CI	Confidence intervals
COX-2	Cyclooxygenase-2
DASH	Dietary approaches to stop hypertension
DSM-5	Diagnostic and statistical manual of mental disorders, fifth edition
ELISA	Enzyme-linked immunosorbent assay
ESS	Epworth sleepiness scale
FFQ	Food frequency questionnaire
GDS-SF	Geriatric depression scale-short form
HDRS	Hamilton depression rating scale
IADL	Instrumental activities of daily living
IL-1α	Interleukin-1α
IL-1β	Interleukin-1β
IL-6	Interleukin-6
iNOS	Inducible nitric oxide synthase
MAPK	Mitogen-activated protein kinase
MCI	Mild cognitive impairment
MDD	Minimum detectable dose
MIND	Mediterranean-DASH intervention for neurodegenerative delay
MMSE	Mini-mental state examination
MoCa	Montreal cognitive assessment
NF-κB	Nuclear factor-kappa B
NO	Nitric oxide
OR	Odds ratio
RCT	Randomized controlled trial
TGF-β1	Transforming growth factor-β 1
TLR4	Toll-like receptor 4
TNF-α	Tumor necrosis factor-α
UPF	Ultra-processed food
