# Supplementary material for: Dietary (Poly)phenol Intake, Inflammatory Biomarkers, and Mild Cognitive Impairment in Italian Adults
Source: Antioxidants (Basel). 2026 Sep 17;15(9):1181. doi: 10.3390/antiox15091181 (PMC13603900; doi:10.3390/antiox15091181)

Supplementary Figure S1. Study selection process.

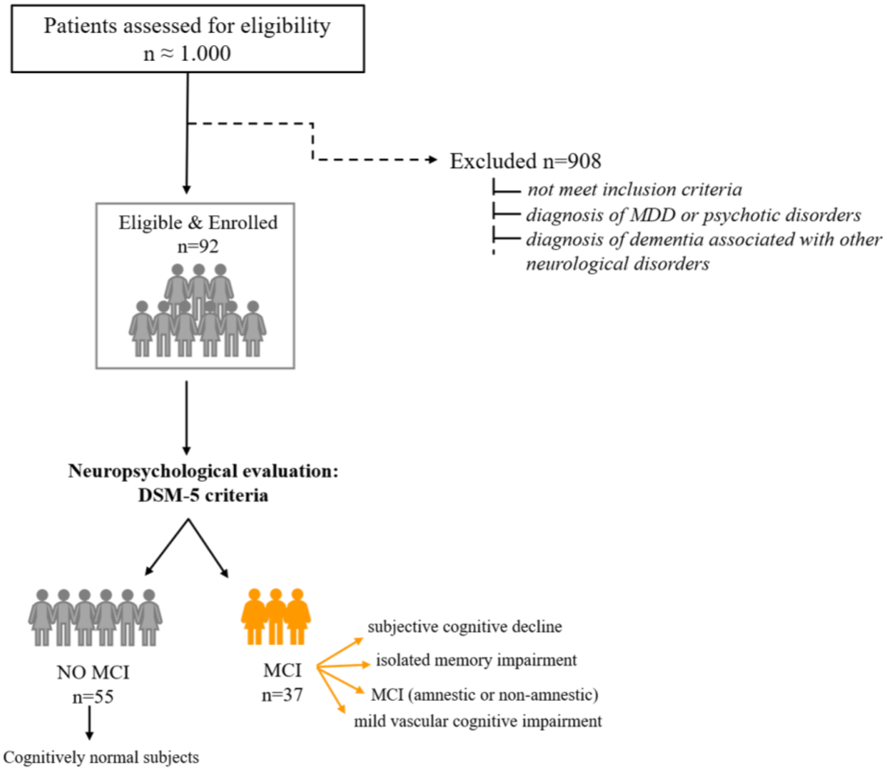

Supplement: Supplementary file 1 [file antioxidants-15-01181-s001.zip › antioxidants-4490589-supplementary.pdf]
